# Multidisciplinary Treatment of Merkel Cell Carcinoma of the Extremities: Outcomes and Factors Associated with Poor Survival in Nodal Disease

**DOI:** 10.3390/curroncol30070462

**Published:** 2023-06-30

**Authors:** Samuel E. Broida, Xiao T. Chen, Brian D. Wahlig, Steven L. Moran, Matthew T. Houdek

**Affiliations:** 1 Department of Orthopedic Surgery, Mayo Clinic, Rochester, MN 55905, USA; broida.samuel@mayo.edu (S.E.B.); chen.xiao@mayo.edu (X.T.C.); wahlig.brian@mayo.edu (B.D.W.); 2 Department of Plastic Surgery, Mayo Clinic, Rochester, MN 55905, USA; moran.steven@mayo.edu

**Keywords:** Merkel cell carcinoma, lymph nodes, immunosuppression, extremity, PET-CT

## Abstract

Merkel cell carcinoma (MCC) has a tendency for lymphatic spread and locoregional recurrence, although there is little data examining the risk factors for patients with lymph node-positive extremity lesions. The purpose of the current study was to examine the outcomes and risk factors associated with nodal metastasis in extremity MCC. We retrospectively reviewed the medical record of 120 patients with extremity MCC evaluated at our institution between 1994 and 2021. The mean age of this cohort was 71 years; 33% of patients were female; and 98% were Caucasian. Seventy-eight (65%) patients presented with localized disease. Thirty-seven (31%) patients had stage III disease, and five (4%) patients had stage IV disease. Treatment of primary lesions consisted primarily of margin-negative excision and adjuvant radiotherapy. Nodal metastases were most treated with adjuvant radiation or completion lymph node dissection. Five-year disease-specific survival in our series was 88% for patients with localized disease, 89% for stage IIIa disease, 40% for stage IIIb disease and 42% for stage IV. Factors associated with worse survival included immunosuppression and macroscopic nodal disease. In conclusion, extremity MCC has a low rate of local recurrence when treated with margin-negative excision and adjuvant radiation. However, treatment of nodal metastases remains a challenge with high rates of recurrence and mortality, particularly for patients who are immunosuppressed or who have macroscopic nodal disease.

## 1. Introduction

Merkel cell carcinoma (MCC) is an uncommon skin malignancy with a propensity for nodal spread and distant metastasis [1]. MCC has a predilection for sun-exposed regions and often affects the extremities [2]. The typical appearance of MCC lesions is as a red or purplish nodule with rapid growth over the course of weeks to months. The presentation of these tumors may be mistaken for other similarly-appearing lesions, such as basal cell carcinoma, squamous cell carcinoma, melanoma or benign entities, such as dermatofibromas or epidermal inclusion cysts. This may lead to misdiagnosis, non-oncologic excision and treatment delays. The aggressive nature of Merkel cell carcinoma necessitates early recognition, proper staging and multidisciplinary treatment [1].

Contemporary guidelines for the management of extremity MCC consist of margin-negative excision, sentinel lymph node biopsy and consideration of radiotherapy for local control [3]. Positive lymph nodes may be treated via additional radiotherapy to the regional basin and/or completion lymph node dissection. Patients with localized MCC have 5-year disease-specific survival rates of 90% through modern approaches to local control [4]. Treatment of node-positive disease has been challenging as 5-year survival rates in this group have been reported to be less than 60% [5,6,7]. Given the dismal prognosis of patients with advanced MCC, it is important to develop a thorough understanding of the demographic, clinical and treatment factors associated with poor prognosis. In addition to adequate staging, identification of at-risk subpopulations of patients with MCC can target patients for whom additional adjuvant or neoadjuvant modalities may be indicated.

There are few reports dedicated to the outcomes and risk factors for recurrence and subsequent mortality in patients with MCC involving the extremities [8]. The aim of this study was therefore to review the outcomes and prognostic factors for patients presenting with extremity MCC, particularly with regard to staging of regional lymph nodes. Our data demonstrate that the distinction between microscopic and macroscopic nodal metastases carries significant prognostic implications for patients with stage III MCC of the extremity.

## 2. Materials and Methods

Following approval from the Institutional Review Board, medical records for all patients evaluated for a diagnosis of MCC between 1994 and 2021 at a single institution. Only patients with a primary cutaneous lesion of the upper or lower extremity, defined as distal to the axillary line or inguinal crease, respectively, were included in this study. All biopsy slides were reviewed by soft tissue pathologists and dermatopathologists at our institution. Patients were excluded if there was not adequate documentation of in-person physical examination or records of evaluation and treatment. A minimum follow-up of 12 months for surviving patients was required for inclusion.

**Figure 1:**
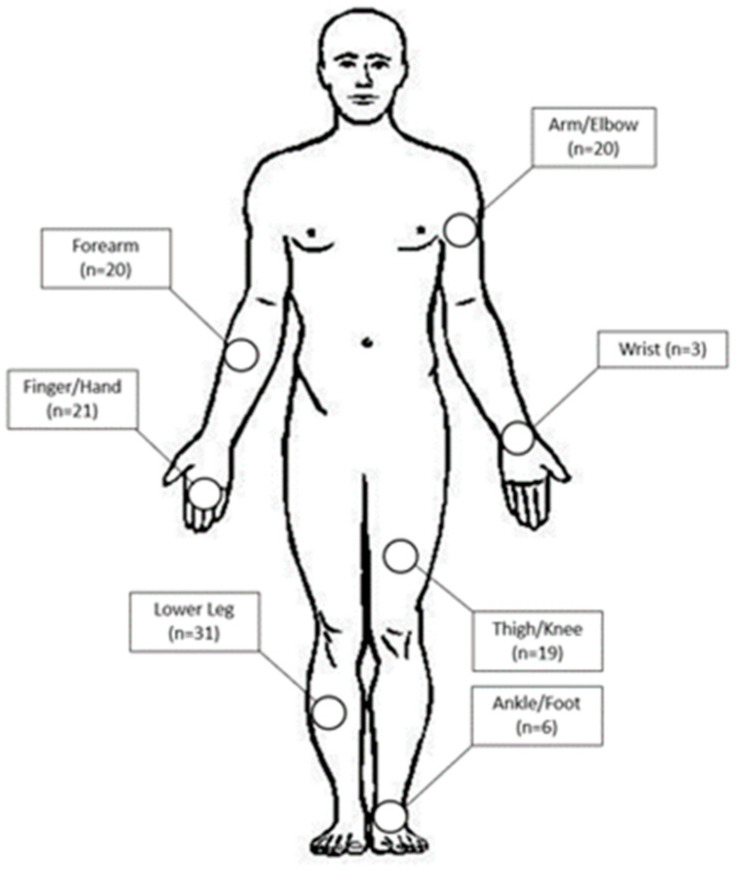
Body map demonstrating locations of primary cutaneous Merkel cell carcinoma lesions of the extremity.

A total of 121 patients were identified. One patient was excluded due to lack of adequate follow-up. Of the 120 patients included in the study, 79 (66%) were male, and 118 (98%) were Caucasian. The mean age at diagnosis was 71 ± 10 years. Primary lesions were located in the upper extremity in 64 patients (53%), and these were identified in the finger/hand (*n* = 21), wrist (*n* = 3), forearm (*n* = 20) or arm/elbow (*n* = 20) (Figure 1). Fifty-six patients (47%) had lesions of the lower extremity, located in the ankle/foot (*n* = 6), lower leg (*n* = 31) and thigh/knee (*n* = 19). Twenty-nine of 120 patients (24%) were immunosuppressed at the time of diagnosis via medications for autoimmune conditions or history of organ transplant.

Local control of non-metastatic tumors consisted of either wide local excision with a goal of 2–3 cm radial margins and the deep margin consisting of fascia, or Mohs’ micrographic surgery. Adjuvant external beam radiotherapy was delivered to the site of the primary lesion in 75% (*n* = 86) of patients with stage I-III disease. Radiation was typically adjuvant and delivered in 2-Gy fractions with total dose ranging from 41 to 66 Gy. Further radiation was administered to the regional lymph node basin in 12% (*n* = 6) of patients with localized disease, 81% (*n* = 17) of patients with stage IIIa disease and 36% (*n* = 5) of patients with stage IIIb disease. Completion lymph node dissection was performed in 37% (*n* = 14) of patients with positive sentinel lymph node biopsy, including 29% (*n* = 6) of patients with stage IIIa disease and 50% (*n* = 7) of patients with stage IIIb disease. Systemic therapy was administered to two patients with metastatic disease (immunotherapy, *n* = 1; carboplatin/etoposide, *n* = 1) and 8 patients with non-metastatic disease (immunotherapy, *n* = 3; carboplatin/etoposide, *n* = 5) who were determined to be high risk for metastasis. Median follow-up for surviving patients was 4 years (range 1–27).

Primary endpoints consisted of recurrence-free survival (RFS) and disease-specific survival (DSS). All chronological endpoints, such as follow-up duration, time without recurrence and duration of survival, were measured from the date of initial diagnosis of Merkel cell carcinoma. Local recurrence was defined as disease recurrence within or adjacent to the operative bed of the primary lesion, whereas regional recurrence was defined as recurrence of disease within the presenting extremity or draining lymph node basin. Distant recurrence was defined as development of new metastatic lesions or recurrence outside of the presenting extremity. All patients were staged according to the American Joint Committee on Cancer staging system (Eighth Edition) [9]. Categorical variables were reported as frequencies and percentages while continuous variables were reported as mean values ± standard deviation (SD). The relationship between clinical or treatment characteristics and survival were analyzed using Cox hazard ratios. Statistical significance was defined as a *p*-value < 0.05. DSS and overall survival estimates were calculated using the Kaplan-Meier method. Statistical analysis was performed via the BlueSky Statistics software package.

## 3. Results

During initial staging, 78 (65%) patients had localized disease. Of these, 54 were stage I lesions, and 24 were stage II. Thirty-seven (31%) patients had stage III disease involving the regional lymph nodes, and the remaining five (4%) patients had stage IV disease.

### 3.1. Lymph Node Investigation

Histopathologic examination of lymph nodes was performed in 109 patients, of which 103 had documented lymph node clinical exams and 57 had PET imaging performed prior to surgery. Histopathologic examination was positive for nodal involvement in 37 (34%) cases. Compared to histopathology for detection of lymph node metastases, clinical examination of lymph nodes had a sensitivity of 35%, specificity of 97%, positive predictive value (PPV) of 86% and negative predictive value (NPV) of 75% whereas PET demonstrated sensitivity of 35%, specificity of 91%, PPV of 73% and NPV of 67% (Table 1). Of patients with confirmed stage III disease and PET imaging or clinical exam, macroscopic nodal disease was absent (stage IIIa) in 21 patients and present (stage IIIb) in 14 patients.

### 3.2. Recurrence

Across all patients, there were four local recurrences. Each patient had previously undergone wide local excision with 2–3 cm radial margins. All four local recurrences were in patients with lower leg primary lesions, and all occurred at the radial margin. One patient had a contaminated wide local excision that was not re-excised prior to local recurrence. Two patients experienced local recurrence despite >2.0 cm tumor-free margins at index wide local excision. Margin data was not available for the fourth patient. All four patients experienced subsequent regional recurrence or progression to metastatic disease, and three of these patients ultimately died of disease at 17, 32 and 47 months from diagnosis.

Twenty-six patients had regional recurrence. These occurred at a median 10 months from diagnosis (range 3–61 months). Neither lymph node dissection nor radiation to the lymph node basin were associated with regional recurrence (*p* = 0.95 and 0.26, respectively). Regional recurrences were treated via surgery (*n* = 7 (27%)), radiation (*n* = 6 (23%)) or surgery and radiation (*n* = 9 (35%)). Systemic therapy was delivered to 12 (55%) patients with regional recurrence. Disease-specific survival from the date of regional recurrence was 96% at 1 year, 71% at 3 years and 64% at 5 years.

Thirty-one patients developed distant metastases following initial treatment. Median time to progression to metastatic disease was 14 months (range 2–118 months) from diagnosis. Distant metastases were treated via surgical resection (20%), radiation (30%) or systemic therapy (70%). Disease-specific survival from the date of metastatic progression was 55% at 1 year, 42% at 3 years and 42% at 5 years.

### 3.3. Survival

DSS for patients with localized (stage I & II) and locoregional (stage III) disease is demonstrated in Table 2. Patients with stage III disease had worse recurrence-free survival compared to those with localized disease (HR 2.42, 95% CI [1.37, 4.30] *p* = 0.002). Among patients with node-negative disease, 5-year RFS was higher in those with upper extremity lesions versus those with lower extremity lesions (76% vs. 56%), but this did not meet statistical significance (*p* = 0.09).

Male gender, age at diagnosis, upper extremity location and primary tumor diameter were not associated with outcome for stage III disease (Table 3). Immunosuppression was not associated with advanced disease stage at diagnosis (OR 1.2, 95% CI [0.50, 2.98]) or recurrence-free and disease-free survival in patients with localized disease (*p* = 0.65). However, immunosuppressed patients with node-positive disease had worse disease-specific survival than those who were not immunosuppressed (HR 3.55, 95% CI [1.14, 11.1], *p* = 0.029).

**Figure 2:**
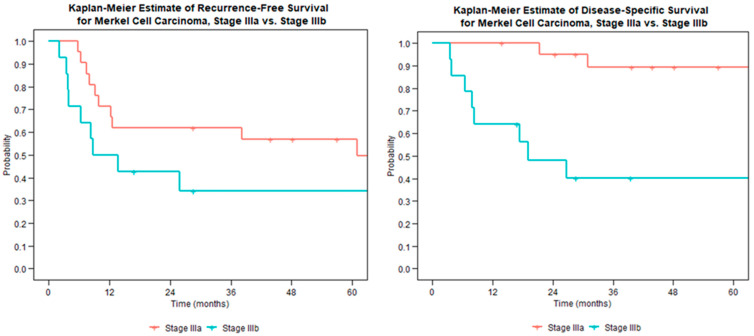
Recurrence-free survival (**left**) and disease-specific survival (**right**) for node-positive Merkel cell carcinoma stratified by microscopic (stage IIIa) or macroscopic (stage IIIb) disease.

The presence of macroscopic nodal disease on imaging or exam was associated with worse survival; however, there was not a significant impact on disease recurrence (Figure 2). Disease-specific survival for stage IIIa tumors was 100% at 1 year and 89% at 5 years. This decreased for stage IIIb disease to 64% at 1 year and 40% at 5 years.

## 4. Discussion

The rarity and aggressiveness of Merkel cell carcinoma makes this disease simultaneously difficult to treat and challenging to study. A thorough understanding of the risk factors associated with poor outcomes can allow for targeted treatment of subgroups at higher risk for disease-specific mortality and recurrence. A previous study out of our institution was one of the first published descriptors of prognostic factors for MCC of the extremities [10]. We sought to build off of these results with an additional 15 years’ worth of patients and follow-up. The results of the present study reflect outcomes of modern treatment approaches and underscore the need for nuanced staging of MCC, particularly with respect to nodal metastases.

The Eighth Edition of the AJCC staging system for Merkel cell carcinoma stratifies stage III disease based on microscopic nodal disease (stage IIIa) versus macroscopic nodal disease that is seen on examination or imaging (stage IIIb). We have previously reported on an additional subset of stage IIIa disease involving patients with macroscopic nodal disease and no identifiable primary lesion [11]; however, the clinical significance of stage IIIa versus stage IIIb in the presence of a cutaneous primary lesion has not been well described. In the present study, we found that the ability to identify nodal disease on examination or PET portends a worse prognosis for patients with stage III MCC. Hypermetabolic activity of a regional node on PET-CT in particular demonstrated a five-fold increase in recurrence and a 25-fold increase in disease-specific mortality. This is likely a reflection of higher tumor burden since larger lesions are more likely to be palpable on examination or visible on imaging. However, our results suggest that more aggressive treatment avenues should be considered for patients who have macroscopic nodal disease. Thorough physical examination and PET imaging are therefore key adjuncts to sentinel lymph node biopsy in the initial staging of patients presenting with cutaneous lesions as they may impact decision-making. While we did demonstrate that examination and imaging are unreliable in the detection of micrometastatic disease, the implications of positive findings on these modalities are important for prognostication and treatment.

Treatment of lymph node metastases in MCC is a source of ongoing debate. While NCCN guidelines recommend adjuvant radiotherapy to the nodal basin and/or completion dissection [9], the optimal approach is still unclear. In our series, there was no difference in recurrence or survival between those who underwent radiation or LND. While the sample size of patients undergoing LND was certainly limited by our institution’s preference towards regional radiation, several prior studies were also unable to detect a difference between adjuvant RT and LND [12,13,14]. On the other hand, a more recent database study of 447 patients with MCC and positive node biopsy found that LND alone was associated with worse overall survival compared with adjuvant RT alone or RT plus LND [15]. Despite the pitfalls of database studies and using overall survival to judge MCC outcomes [4], the aforementioned study remains the most compelling evidence to date regarding the inclusion of radiotherapy in treatment of nodal metastases. It is unlikely that powered prospective trials can be done, the treatment of regionally advanced MCC should be individualized through collaborative discussion between medical and radiation oncologists as well as surgeons [16].

Merkel cell carcinoma has been linked to immunosuppression previously; however, studies on this association have mostly centered on the impact of immunosuppression on the frequency of MCC diagnosis rather than prognosis. Recent studies out of the United States and Ireland have shown that the risk of MCC in organ transplant recipients is increased 24-fold and 60-fold, respectively [13,17]. In our series, immunosuppression was not associated with increased recurrence or mortality for localized disease; however, immunosuppression was an independent risk factor for recurrence and mortality in patients who had stage III disease. A recent paper comparing patients with intact and compromised immune systems found worse survival regardless of stage [18]. This study did not report on methods of treatment for patients in the cohort; we attribute the differential impact of immunosuppression on localized versus regionally advanced tumors attributable to the overall success of the modern approach to local tumor control, namely margin-negative excision with adjuvant radiation therapy. As the role of Merkel cell polyomavirus in oncogenesis and tumor progression becomes more clear [19], targeted studies on the optimal treatment and surveillance of immunosuppressed patients will be necessary.

In the 15 years since Senchenkov et al. published their examination of treatment approaches and associated factors for recurrence and survival of extremity MCC [10], adjuvant radiation therapy has become more of a mainstay of treatment for locoregional disease [20]. In this updated series, radiotherapy was delivered to a larger proportion of patients with localized disease and nodal disease, including 80% of patients who were included since 2007. This likely accounts for the improvement in disease survival in the interim. Five-year disease-specific survival for localized disease approached 90%, nearly the same as for stage IIIa patients. This same metric dropped to 40% for stage IIIb disease. The arrival of pembrolizumab and other immunomodulators has altered the approach to disseminated disease [21]. In our study, immunotherapy was almost exclusively used for recurrent or metastatic disease, and therefore, we considered its effects to be beyond the scope of our analysis. In light of our poor outcomes for patients with macroscopic nodal disease, immunotherapy should be considered in this population.

Though the NCCN treatment guidelines provide a standard approach to management of MCC [3], the poor survival of stage IIIb patients in this cohort highlight the need for continued advancements in treatment. Further large, prospective and randomized studies are needed to identify whether diseased lymph nodes are best treated via completion dissection versus radiotherapy, or both. As immunotherapy becomes more common and accessible, investigation into the outcomes of patients who receive these medications for initial treatment of advanced disease is needed. An understanding of these adjuvant modalities would allow for refinement of current guidelines to provide a targeted multidisciplinary approach to the high risk subpopulations of patients with extremity MCC.

This study may be viewed in light of several limitations. All data was collected at a large, tertiary referral center and therefore may not fully represent the whole population of patients with this disease. Patients were treated over a 25-year period, during which time the staging and treatment of Merkel cell carcinoma has slowly shifted. A diverse group of clinicians, including dermatologists, oncologists, radiation oncologists, surgical oncologists and orthopedic oncologists, may have introduced additional heterogeneity into decision-making and treatment courses. Additionally, the cohort involves a relatively small number of patients on whom data was retrospectively obtained, and confounding demographic, staging or treatment characteristics were unable to be controlled for. As a result, multivariable analysis was not possible and determining why patients received one treatment over another is not always clearly stated. However, this study represents the largest single-institution cohort of patients with extremity MCC in the current literature.

## 5. Conclusions

MCC is a rare and aggressive cutaneous neoplasm with a tendency to metastasize to regional lymph nodes and distant sites. In our series of patients with MCC of the extremity, we observed a high rate of 5-year disease-specific survivorship in patients with localized tumors; however, recurrence and disease-specific mortality are high for patients with nodal disease. This is especially pronounced when nodal metastases are detectable on clinical examination or PET-CT. These modalities are therefore key for appropriate staging of all patients with risk factors for nodal disease, such as clinically advanced extremity lesions. We recommend multidisciplinary treatment approaches to all tumors with wide local excision and radiation therapy in all local and locally advanced tumors. Patients with macroscopic nodal metastases may benefit from more aggressive treatment and surveillance. Future investigations are needed into the role of immunotherapy in the initial treatment of patients with regional metastases, particularly for those with stage IIIb disease.

## Figures and Tables

**Table 1:** Performance of clinical exam and PET imaging compared to gold standard of histopathology in detection of regional nodal involvement in patients with MCC of the extremity.

Exam Modality	Sensitivity	Specificity	PPV	NPV
Clinical Exam (*n* = 103)	35% (12/34)	97% (67/69)	86% (12/14)	75% (67/89)
FDG-PET (*n* = 57)	35% (8/23)	91% (31/34)	73% (8/11)	67% (31/46)

**Table 2:** Cox Hazard Analysis for Risk Factors Associated with Recurrence-Free Survival and Disease-Specific Survival with Stage III MCC.

	Recurrence-Free Survival			Disease-Specific Survival		
	HR	95% CI	*p*-Value	HR	95% CI	*p*-Value
Male gender	2.0	[0.8, 5.2]	0.14	2.5	[0.7, 9.4]	0.16
Age at Diagnosis	1.1	[1.0, 1.1]	0.05	1.1	[1.0, 1.1]	0.06
Upper Extremity Location	1.4	[0.6, 3.2]	0.44	1.7	[0.6, 5.3]	0.35
Max Diameter	1.1	[0.9, 1.4]	0.68	1.1	[0.8, 1.7]	0.50
Immunosuppressed	2.1	[0.9, 4.9]	0.10	3.6	[1.1, 11]	0.03 *
Macroscopically-Positive Node	2.0	[0.8, 4.7]	0.12	5.7	[1.7, 19]	<0.01 **
Palpable Lymphadenopathy	2.4	[0.9, 6.2]	0.07	5.1	[1.6, 17]	<0.01 **
Hypermetabolic Node on PET	5.0	[1.4, 18]	<0.01 **	24.7	[2.8, 220]	<0.01 **
Complete Node Dissection	1.1	[0.5, 2.5]	0.90	1.6	[0.5, 4.8]	0.40
Radiation to Primary Site	0.6	[0.1, 2.6]	0.48	1.2	[0.2, 10]	0.84
Radiation to Lymph Nodes	1.1	[0.4, 3.3]	0.90	0.8	[0.2, 3.0]	0.74
XRT alone (ref. RLND alone)	1.0	[0.4, 2.6]	0.95	0.7	[0.2, 2.3]	0.54
Systemic Therapy	0.7	[0.2, 2.3]	0.52	0.5	[0.1, 3.6]	0.46

HR: Hazard Ratio; * *p* < 0.05; ** *p* < 0.01.

**Table 3:** Disease recurrence and disease-specific survival for patients with Merkel cell carcinoma of the extremity.

		Disease Recurrence				Disease-Specific Survival		
	Total *n*.	Loc.	Reg.	Dist.	None	1 Year	3 Years	5 Years
**Stage I&II Disease**	78	3	16	13	54	97%	92%	88%
Wide local excision	70	3	14	12	49	97%	91%	86%
Adjuvant RT to primary	51	1	10	9	36	96%	94%	90%
Adjuvant RT to LN	9	1	2	4	6	100%	89%	89%
(+) Adjuvant Chemo	3	0	1	0	2	100%	100%	100%
**Stage III Disease**	38	1	10	17	17	86%	71%	71%
Wide local excision	34	1	10	14	16	91%	73%	73%
Therapeutic LND	14	1	3	7	4	94%	59%	59%
Adjuvant RT to primary	33	1	10	15	13	88%	74%	74%
Adjuvant RT to LN	29	1	9	12	14	86%	74%	74%
(+) Adjuvant Chemo	6	0	2	2	3	83%	83%	83%
**All Stages**	120	4	26	31	70	93%	84%	82%
Upper extremity	64	0	11	12	41	94%	82%	82%
Lower extremity	56	4	15	19	29	91%	87%	80%

RT: radiotherapy; LN: lymph node.

## Data Availability

The data presented in this study are available on request from the corresponding author. The data are not publicly available due to privacy restrictions.

## Abbreviations

MCC	Merkel cell carcinoma
RFS	recurrence-free survival
DSS	disease-specific survival
PET	positron emission tomography
PPV	positive predictive value
NPV	negative predictive value
HR	hazard radio
CI	confidence interval
RT	Radiotherapy
LN	lymph node
LND	lymph node dissection
AJCC	American Joint Committee on Cancer
